# Diagnostic accuracy of chest ultrasound in patients with pneumonia in the intensive care unit: A single‐hospital study

**DOI:** 10.1002/hsr2.102

**Published:** 2018-11-26

**Authors:** Zouheir Ibrahim Bitar, Ossama Sajeh Maadarani, AlAsmar Mohammed El‐Shably, Mubarak Juwaied Al‐Ajmi

**Affiliations:** ^1^ Internal Medicine Department Ahmadi Hospital, Kuwait Oil Company Ahmadi Kuwait; ^2^ Internal Medical Department Ahmadi Hospital Ahmadi Kuwait; ^3^ Respiratory Unit Ahmadi Hospital Ahmadi Kuwait

**Keywords:** lung ultrasound, pneumonia

## Abstract

**Background and aims:**

Chest radiography (CXR) and computerized tomography (CT) scan are the preferred methods for lung imaging in diagnosing pneumonia in the intensive care unit, in spite of their limitations. The aim of this study was to assess the performance of bedside lung ultrasound examination by a critical care physician, compared with CXR and chest CT, in the diagnosis of acute pneumonia in the ICU.

**Materials and Methods:**

This was an observational, prospective, single‐center study conducted in the intensive care unit of Ahmadi General Hospital. Lung ultrasound examinations (LUSs) were performed by trained critical care physicians, and a chest radiograph was interpreted by another critical care physician blinded to the LUS results. CT scans were obtained when clinically indicated by the senior physician.

**Results:**

Out of 92 patients with suspected pneumonia, 73 (79.3%) were confirmed to have a diagnosis of pneumonia based on radiological reports, clinical progress, inflammatory markers, and microbiology studies. Of the 73 patients, 31 (42.5%) were male, with a mean age of 68.3 years, and a range of 27 to 94 years. Eleven (15%) patients had community‐acquired pneumonia, and 62 (85%) had hospital‐acquired pneumonia. In the group of patients with confirmed pneumonia, 72 (98.6%) had LUSs positive for consolidation (sensitivity 98.6%, 95% CI 92.60%‐99.97%), and in the group without pneumonia, 16 (85%) had LUS negative for consolidation (specificity 84.2%, 95% CI 60.42%‐96.62%), compared with 40 (55%) with CXRs positive for consolidation (sensitivity 54.8%, 95% CI 42.70%‐66.48%) and 33 (45%) with CXRs negative for consolidation (specificity 63.16%, 95% CI 38.36%‐83.71%).

A chest CT was performed in 38 of the 92 enrolled patients and was diagnostic for pneumonia in 32 cases. LUSs were positive in 31 of 32 patients with CT‐confirmed pneumonia (sensitivity 96%), and CXR was positive in 5 of 32 patients with CT‐confirmed pneumonia (sensitivity 15.6%).

**Conclusion:**

Bedside lung ultrasound is a reliable and accurate tool that appears to be superior to CXR for diagnosing pneumonia in the ICU setting. LUS allows for a faster, non‐invasive, and radiation‐free method to diagnose pneumonia in the ICU.

## 1 INTRODUCTION

Pneumonia is still a major health care problem, with an important effect on mortality and morbidity worldwide.^1^ Pneumonia affects 5.16 to 7.06 people per 1000 person‐years, and the rate of pneumonia increases with increasing age.^2^ Pneumonia is the second‐most common type of nosocomial infection of high mortality and is considered an important health care‐related complication.^3^

Diagnosing pneumonia is challenging to physicians, as there is wide variety of conditions to consider in its differential diagnosis, eg, cardiogenic and non‐cardiogenic pulmonary edema, malignancy, hemorrhage, pulmonary embolism, and inflammation secondary to noninfectious causes.^4^

The presence of a set of suggestive clinical features and microbiological testing results, along with the presence of consolidation or opacity on chest radiography (CXR) or computerized tomography (CT) scan of the chest, confirms the diagnosis of pneumonia.^5^, ^6^ A CXR is the standard test for evaluation of a patient with suspected pneumonia, as medical history and physical examinations cannot provide certitude in diagnosing pneumonia.^7^ For hospitalized patients with suspected pneumonia and a negative CXR, the 2007 IDSA/ATS consensus guidelines consider it reasonable to initiate empiric presumptive antibiotic therapy and repeat the chest radiograph in 24 to 48 hours.^5^

A chest CT scan is performed in clinically suspected pneumonia patients with a non‐conclusive CXR. A chest CT scan is more informative than plain films for the evaluation of various lung diseases such as interstitial lung disease, cavitation, septations in empyema, and diffuse infiltrative diseases.^8^ A CT scan in community acquired pneumonia is more sensitive than CXR, but it is not recommended for routine use because of its high cost and no associated improvement in outcomes.^5^

There is increasing interest in the use of lung ultrasound (LUS) to diagnose pneumonia, particularly in unstable patients in the emergency department or intensive care unit (ICU) where it is difficult to obtain a quality CXR. In a meta‐analysis of 12 trials, lung ultrasound had a sensitivity of 88% and a specificity of 86% when compared with CXR or chest CT.^9^ It may thus be reasonable for those with experience in performing lung ultrasounds to use this modality when a reasonable quality CXR cannot be obtained. Real‐time imaging, better portability, the ability to perform dynamic imaging, and the absence of radiation are advantages of lung ultrasound over traditional radiographic imaging of the pleura.^9^

The purpose of this study was to assess performance of bedside lung ultrasounds as a first line diagnostic test to confirm the diagnosis of pneumonia in patients with clinical suspicion for pneumonia in the ICU compared with traditional standard radiological tests.

## 2 METHODS

The study was conducted in Ahmadi hospital medical‐surgical ICU, Kuwait, between December 2015 and April 2017. The study was approved by the Ahmadi Hospital Ethics Committee, and informed consent was obtained from every patient or next of kin.

Patients with suspicion of pneumonia and sepsis consecutively admitted to our ICU from the ED or transferred from general wards, and patient on ventilators with signs of pneumonia, were included.

In patients with clinical histories of cough and sputum production, fever, pleuritic chest pain, dyspnea, and physical examinations suggestive of pneumonia (temperature > 38°C or less than 36°C, respiratory rate > 22 breaths/minute, heart rate > 90/minute, and chest examination revealing audible crackles, decreased or bronchial breath sounds, dullness to percussion, or tactile fremitus), LUSs were performed by a trained intensivist. CXR was consistently performed after LUS. A positive CXR confirmed the diagnosis of pneumonia, irrespective of LUS results. On the other hand, cases with a positive LUS and a negative CXR were evaluated by CT according to preexisting protocols. CXR and CT scans were interpreted by the on duty senior radiologist, who was aware of the clinical suspicion for pneumonia but not of the LUS results. We conducted a 10‐day follow‐up in patients with confirmed pneumonia, to verify clinical and laboratory progress with CXR and LUS for lung reaeration following antibiotic therapy. We obtained an additional separate informed consent only from patients undergoing CT scan because LUS examination and CXR are considered routine procedures, while a CT scan is not, because of the high radiation, and, in agreement with other studies,^7^ we considered that performing it for all patients was not ethically justified.

As a rule, community‐acquired pneumonia refers to that present on hospital admission or that occurs within the first 48 hours after admission, indicating that there was already incubation at the time of admission. In contrast, according to the American Thoracic Society (ATS) guidelines, health care‐associated pneumonias are defined as those occurring after 48 hours or more following admission to a health care facility, implying that there was no incubation at the time of admission.^4^ Ventilator‐associated pneumonia (VAP) refers to that which develops in ICU patients who have been mechanically ventilated for at least 48 hours.

The diagnosis of pneumonia was confirmed by a set of clinical features (clinical history and physical examination), microbiological testing for admitted patients (blood and sputum culture, legionella and pneumococcal urinary antigen testing, and multiplex polymerase chain reaction assay for detecting *Chlamydia pneumoniae*, *Mycoplasma pneumoniae*, and respiratory tract viruses), inflammatory markers (c‐reactive protein >10 mg/L and procalcitonin ≥0.25 ng/mL), along with the presence of consolidation or opacification on a CXR or chest CT.^5^, ^6^ Children (<16 years old) and pregnant women were excluded because of the restrictions for the use of CT in these patients.

All included patients were prospectively evaluated until discharge. The final diagnosis was made by the physician in charge, based on radiological reports, clinical progress, inflammatory markers, and microbiology studies. We compared the ultrasound results with the final diagnosis made by physicians in charge. We also compared the result of CT chest and LUS with the final diagnosis made by the treating physician.

A phased‐array 5‐MHz ultrasound probe (GE Vivid S6N, N‐3191 Horten, Norway) was used. At the bedside, the probe was set perpendicular, oblique, and parallel to the ribs in the anterior, lateral, and posterior (lower and upper) thorax, respectively. The sitting and lateral decubitus positions were used to scan the posterior chest wall.

In accordance with the literature,^10^, ^11^, ^12^ we evaluated 10 chest areas, five in each hemithorax: two anterior, two laterals, and one posterior in each side. The anterior chest wall was defined from the parasternal line to the anterior axillary line. This zone was divided into upper and lower regions, at third intercostal space. The lateral area from the anterior to the posterior axillary line was divided into the upper and lower halves. The posterior zone was identified from the posterior axillary line to the paravertebral line.

**Figure 1:**
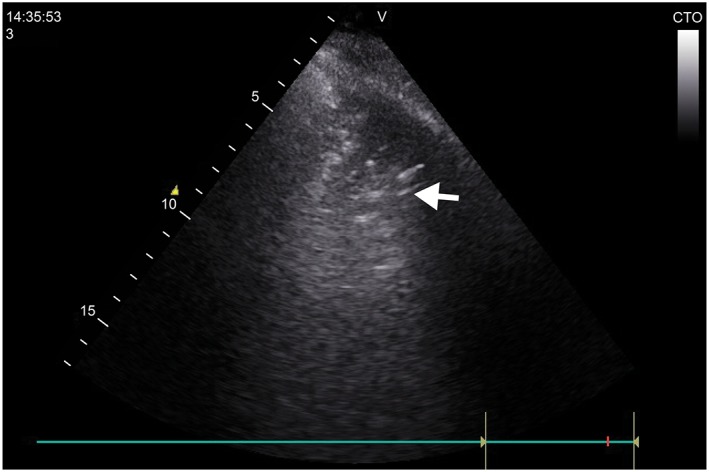
Ultrasound image. A consolidated right lung on ultrasound in a case of pneumonia

The healthy lung displays pleural sliding and A‐lines (repetitive lines parallel to the pleural line) on ultrasound.^13^ In interstitial syndrome, there are multiple B lines (more than three lines in one region). A B line is defined as a long vertical hyperechoic reverberation artifact that arises from the pleural line extending to the bottom of the screen that fades and moves synchronously with pleural sliding.^13^ Ultrasonic diagnosis of pneumonia depends on the presence of lung consolidation attaching to the pleural (subpleural), presenting tissue‐like pattern (due to decrease alveolar aeration and increase fluid content) (Figure 1).^14^ The boundaries of the consolidated areas are the normal aerated lung and the pleura. In real time ultrasound, the consolidations may contain dynamic air bronchograms (branching echogenic structures with scattered air artefacts moves through the bronchi with breathing) or multiple hyperechogenic spots (air trapped in the small airway).^14^ The presence of a focal interstitial syndrome (focal distribution of B lines) is considered evidence of pneumonia.^13^ Pleural effusion is an anechoic space between the parietal and visceral pleura with respiratory movement of the lung within the effusion.^13^

Statistical analyses were performed using SPSS 19. Sensitivity, specificity, and positive and negative likelihood ratios of lung ultrasound and CXR for the diagnosis of pneumonia were calculated. The McNemar test was used for dicotomic variables when appropriate. *P* value was considered significant if <0.05.

## 3 RESULTS

Out of 92 patients with a suspicion of pneumonia that were admitted to the ICU, 73 were confirmed to have a diagnosis of pneumonia (79.3%, see methods). Among them, there were 31 (42.5%) males, and the mean age was 68.3 years, with a range of 27 to 94 years. Eleven (15%) patients had community‐acquired pneumonia, and 64 (85%) had hospital‐acquired pneumonia. In the group of patients with confirmed pneumonia, 72 (98.6%) and 40 (54.8%) cases were positive for consolidation on LUS and CXR, respectively (Table 1). The specificity and sensitivity of CXR and LUS are shown in Table 2. There was overall concordance between the findings of consolidation on LUS and CXR (k statistic = 0.63; 95% confidence interval, 0.43‐0.84), but the number of cases with positive LUS and negative CXR was significantly greater than the number of patients with negative LUS and positive CXR (105 vs 41, *P* = 0.0196, McNemar test).

**Table 1:** Lung ultrasound and CXR profiles based on diagnosis of pneumonia (*N* = 92)

Diagnostic Tool	Confirmed Bronchopneumonia (*N* = 73)		Sensitivity, %	Specificity, %	PPV, %	NPP, %	PLR, %	NLR, %
	Present	Absent						
LUS								
LU+	72	3	98.63	84.21	96	94	6.25	0.02
LU−	1	16						
CXR								
CXR+	40	7	54.79	63.16	85	26.67	1.49	0.72
CXR−	33	12						

All the patients underwent lung ultrasound (LU) first then chest X‐ray (CXR), positive (+) or negative (−) for the abnormality; bronchopneumonia present or absent based on final diagnosis; TP, true positive; TN, true negative; FP, false positive; FN, false negative; PPV, positive predictive value; NPV, negative predictive value; PRL, positive likelihood ratio; NLR, negative likelihood ratio.

**Table 2:** Ultrasound and CXR compared with CT (*N* = 38)

Diagnostic Tool	CT Chest with Confirmed Pneumonia (*N* = 32)		Sensitivity, %	Specificity, %	PPV, %	NPP, %	PLR, %	NLR, %
	Positive	Negative						
LUS								
LU+	31	1	96.9	83	97	83	5.81	0.04
LU−	1	5						
CXR								
CXR+	5	5	15.6	16.67	50	3	0.19	5
CXR−	27	1						

LU, lung ultrasound; CXR, chest X‐ray; positive (+) or negative (−) for the abnormality; bronchopneumonia present or absent based on final diagnosis; TP, true positive; TN, true negative; FP, false positive; FN, false negative; PPV, positive predictive value; NPV, negative predictive value; PRL, positive likelihood ratio; NLR, negative likelihood ratio.

A chest CT was performed in 38 of the 92 enrolled patients and was diagnostic for pneumonia in 32 cases. In the other six cases, chest CT showed one case of pulmonary adenocarcinoma, one case of metastatic cancer breast, one case of granulomatosis with polyangiitis, one case of pulmonary embolism, and two cases of acute respiratory distress syndrome. LUS was positive in 31 of 32 patients with CT‐confirmed pneumonia, and CXR was positive in five of 32 patients with CT‐confirmed pneumonia. The sensitivity and specificity of LUS and CXR compared with those of the CT scan are shown (Table 2).

**Figure 2:**
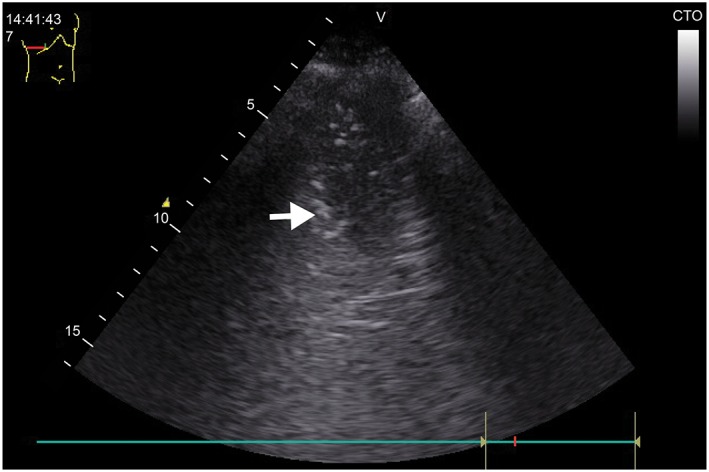
Ultrasound image. Alveolar consolidation with a frank tissue pattern arising from the pleural line with an irregular, shredded border (arrows). The shred sign is seen because the consolidation is in contact with the aerated lung

**Figure 3:**
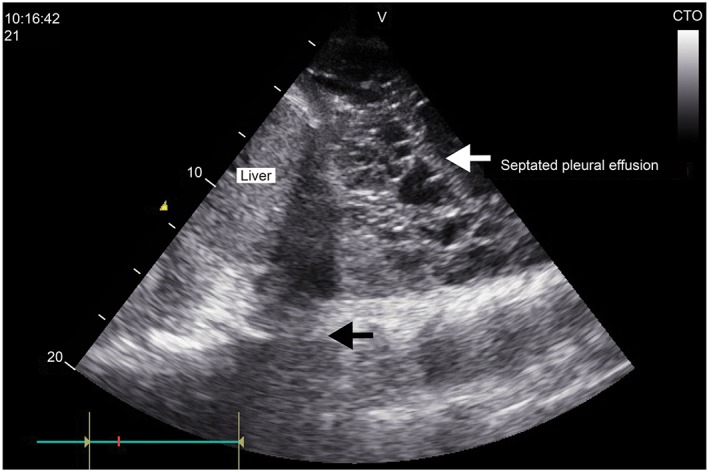
Ultrasound image. Complex pleural effusion with multiple septations. PE pleural effusion

Complete LUSs (scanning the anterior, lateral, and posterior chest walls) were performed in the 92 recruited patients and took 5 minutes on average. The ultrasound findings in patients with pneumonia and positive LUS are summarized in Table 3. Examples of consolidation on ultrasound are shown in Figures 1 and 2. In patients with pneumonia and positive LUS, a significant reaeration was observed at day 10 by LUS, together with a partial improvement or a complete disappearance of ultrasonic consolidation noticed in 38 cases (52%). Complicated effusions were detected in three cases (Figure 3). In these cases, empyema was suspected early on LUS, and a pigtail catheter was inserted under ultrasound guidance. One case showed multiple encysted effusions on ultrasound, and two pigtail catheters were inserted.

**Table 3:** LUS findings in patients with pneumonia and positive LUS [*N* = 72]

LUS Findings in Pneumonia	Frequency, %
Interstitial syndrome	11(15)
Consolidation	61(85)
Effusion	23(31.5)
Complicated effusion	3 (4)
Dynamic air bronchogram	58 (79)

A patient with positive LUS could have more than one ultrasonic findings of pneumonia.

In all included cases (*N* = 92), portable antero‐posterior CXRs were performed without lateral views. Portable lateral view CXRs are difficult to obtain and are not routinely practiced in the ICU. We obtained a repeat CXRs after 48 hours as recommended in the guidelines for non‐diagnostic radiology.^15^

## 4 DISCUSSION

Diagnosing pneumonia in the ICU is challenging. Pneumonia is a complex disease consisting of both community‐acquired and nosocomial pneumonias, most commonly VAP, which have common radiological findings.^4^

LUS has been shown to be an important tool in the hands of critical care physicians for the diagnosis of pneumothorax, acute pulmonary edema, pleural effusions, and other pulmonary diseases.^16^ The use of LUS in the diagnosis and follow‐up of pneumonia has also been investigated in view of the limitations of CXR.^17^ The findings of our study are consistent with those of others showing that LUS is superior to CXR as diagnostic tool in pneumonia cases (17). In this study, the gold standard chest CT was carried out in a greater number of patients compared with other studies,^14^ and the decision to obtain a chest CT was made by the treating critical care physician. The inferiority of CXR observed in this study may be due in part to the inability to perform lateral view CXRs. The limitation of CXR will be clear when performed for critically ill patients in the ICU and ED, as examination will only be in the supine position, often with a bedside machine.^18^ In the ED, it was found that a bedside LUS is a valid tool for the detection of consolidations, with superiority to CXR. The study concluded that broader use of lung ultrasound will allow for more timely diagnosis and implementation of appropriate therapy.^19^ A meta‐analysis of published data has also shown that LUS is a valid bedside exam to diagnose and monitor VAP, especially in the intensive care setting, and reduces patients' exposure to radiation.^20^

The diagnostic sign of pneumonia on ultrasound is the presence of consolidation and interstitial syndrome, which is nonspecific.^16^ Consolidation, identified in most of our cases, can also be present in obstructive atelectasis. Dynamic air bronchograms observed within the consolidation due to pneumonia can easily be detected by ultrasound and help differentiate pneumonia from atelectasis with high sensitivity and specificity.^21^ This dynamic sign on LUS is an advantage over CXR, and even CT images. Dynamic air bronchograms were present in 58 out of the 73 patients with confirmed pneumonia. Bronchoscopy examination is indicated when static air bronchograms are detected by LUS within a large pneumonic consolidation in association with the “lung pulse” sign and decreased pleural sliding.^22^ Interstitial syndrome pattern was detected in a small number of patients with confirmed pneumonia in our study. The ultrasonic signs of focal interstitial syndrome suggest pneumonia, but in cases of bilateral interstitial syndrome of acute onset it suggests pulmonary edema or acute lung injury/acute respiratory distress syndrome.^13^ We observed four patients with bilateral interstitial syndrome in our study, and because of the presence of small subpleural consolidations, the decreased or absent pleural sliding movement, and the irregularity and thickening of the pleural lines, we excluded pulmonary edema. The ultrasound features in these four patients were like those described by Volpicelli et al^12^ in acute lung injury/acute respiratory distress syndrome; the final diagnosis of lung infection was supported by the microbiological results.

Detecting the nature of effusions by chest US was an advantage over CXR, as detecting echoes within the effusion, whether mobile particles or septa, is suggestive of exudate.^13^ In our study, complicated effusions were detected by chest US and followed by ultrasound‐guided chest tube insertions.

### 4.1 Limitations

The current study has a relatively small sample size and was conducted in a single center. Chest CT was carried out in a restricted number of patients, in a nonrandomized manner. The advantage of LUS over CXR was, therefore, confirmed with reference to the hospital discharge diagnosis of pneumonia and not with the gold standard CT chest test. In agreement with other studies,^7^ we agreed that exposing all patients to CT scans radiations would not be ethically justified.

## 5 CONCLUSION

In conclusion, bedside LUS is an accurate and reliable tool for detecting pneumonia in the critical care setting, displaying superiority to CXR. It is a timely, non‐invasive, and radiation‐free modality for the diagnosis of pneumonia in the ICU.

## FUNDING SOURCES

This study was funded by Kuwait Oil Company, Ahmadi Hospital. The funders had no role in study design, data collection and analysis, interpretation of data, or preparation of the manuscript.

## CONFLICTS OF INTEREST

None declared.

## AUTHOR CONTRIBUTIONS

Conceptualization: Zouheir Bitar Ossama Maadarani, Mubarak Al‐Ajmi

Formal analysis: Zouheir Bitar, AlAsmar El‐Shably

Writing—original draft: Zouheir Bitar

Writing—review and editing: Zouheir Bitar, Ossama Maadarani, AlAsmar El‐Shably, Mubarak Al‐Ajmi

The authors had full access to all the data in this study and take complete responsibility for the integrity of the data and the accuracy of the data analysis.
